# Growth dynamics of lung nodules: implications for classification in lung cancer screening

**DOI:** 10.1186/s40644-024-00755-y

**Published:** 2024-08-26

**Authors:** Beatriz Ocaña-Tienda, Alba Eroles-Simó, Julián Pérez-Beteta, Estanislao Arana, Víctor M. Pérez-García

**Affiliations:** 1 Mathematical Oncology Laboratory, University of Castilla-La Mancha, Ciudad Real, Spain; 2 Instituto de Instrumentación para la Imagen Molecular (i3M), Universitat Politécnica de València, Consejo Superior de Investigaciones Científicas (CSIC), València, Spain; 3 Department of Radiology, Fundación Instituto Valenciano de Oncología, Valencia, Spain

**Keywords:** CT, Growth dynamics, Lung cancer, Lung nodules, Screening

## Abstract

**Background:**

Lung nodules observed in cancer screening are believed to grow exponentially, and their associated volume doubling time (VDT) has been proposed for nodule classification. This retrospective study aimed to elucidate the growth dynamics of lung nodules and determine the best classification as either benign or malignant.

**Methods:**

Data were analyzed from 180 participants (73.7% male) enrolled in the I-ELCAP screening program (140 primary lung cancer and 40 benign) with three or more annual CT examinations before resection. Attenuation, volume, mass and growth patterns (decelerated, linear, subexponential, exponential and accelerated) were assessed and compared as classification methods.

**Results:**

Most lung cancers (83/140) and few benign nodules (11/40) exhibited an accelerated, faster than exponential, growth pattern. Half (50%) of the benign nodules versus 26.4% of the malignant ones displayed decelerated growth. Differences in growth patterns allowed nodule malignancy to be classified, the most effective individual variable being the increase in volume between two-year-interval scans (ROC-AUC = 0.871). The same metric on the first two follow-ups yielded an AUC value of 0.769. Further classification into solid, part-solid or non-solid, improved results (ROC-AUC of 0.813 in the first year and 0.897 in the second year).

**Conclusions:**

In our dataset, most lung cancers exhibited accelerated growth in contrast to their benign counterparts. A measure of volumetric growth allowed discrimination between benign and malignant nodules. Its classification power increased when adding information on nodule compactness. The combination of these two meaningful and easily obtained variables could be used to assess malignancy of lung cancer nodules.

**Supplementary Information:**

The online version contains supplementary material available at 10.1186/s40644-024-00755-y.

## Background

Lung cancer is the leading cause of cancer-related mortality, exceeding deaths from breast, prostate, and colorectal cancers combined [1, 2]. Lung cancer incidence has steadily decreased since 2006–2007, and these reductions are not only due to improved therapies [1, 3] but also to advances in early detection [1], staging [4], and surgical techniques [5]. The effectiveness of lung cancer screening (LCS) has been demonstrated with a proven 20-year survival rate of 81% [1].

Investigation of lung-tumor growth patterns provides valuable insights into its underlying biology. Clinically, understanding the in vivo growth rate is essential for establishing appropriate diagnosis and treatment intervals. Additionally, knowledge of the growth dynamics of pulmonary nodules plays a critical role in their differentiation. Currently, the guidelines for managing pulmonary nodules assume exponential growth in distinguishing between malignant and benign nodules while monitoring them [6]. However, this assumption is not consistent across studies. While some authors argue that the growth of lung nodules follows an exponential course [7–9], others state that it follows neither exponential, nor linear, nor Gompertzian growth patterns [10, 11]. Lindell et al. [11]. observed that growth curves exhibited an accelerated pattern, characterized by growth rates steeper than those of traditional exponential models. This observation aligns with the finding that non-small-cell lung cancer (NSCLC) longitudinal growth dynamics are faster than exponential [12].

The study had two goals. Firstly, it aimed to investigate the growth dynamics of lung nodules. Secondly, it tried to compare different methods for distinguishing lung cancer from benign cases by assessing low-dose CT scans.

## Methods

### Study participants

This retrospective study analyzed data from an institutional Review Board-approved screening program with a waiver of informed patient consent, following the International Early Lung Cancer Action Program [13] (I-ELCAP) protocol, as I-IELCAP screening site (Valencian Institute of Oncology, Valencia, Spain) [14]. The program targets individuals aged 50 or older, in good health, asymptomatic, with a smoking history of at least 30 packs per year. Participants may also include those with a smoking history of 20–30 packs, additional risk factors, or former smokers who have stopped for less than 15 years. The I-ELCAP protocol involves three stages: initial nodule detection through CT scans, ongoing monitoring of nodule growth (CT-based), and conclusive detection through histopathologic confirmation.

Chronic obstructive pulmonary disease (COPD) was classified by spirometry as mild (forced expiratory volume in 1 s FEV1 ≥ 80% predicted), moderate (80% > FEV1 ≥ 50%), severe (50% > FEV1 ≥ 30%), and very severe (FEV1 < 30%), according to Global Initiative for Chronic Obstructive Lung Disease criteria [15].

Screening was performed between June 2008 and November 2020. Among 8278 participants, 180 participants met inclusion criteria, comprising 140 (77.8%) diagnosed with lung tumors and 40 (22.2%) with benign, progressively growing nodules. The inclusion criteria for this study imposed having a minimum of three available follow-ups with noticeable size increases and available histopathologic confirmation.

### Data acquisition

All CT scans were performed on two scanners; 100 subjects on a 16-slice multi-detector spiral CT (Siemens Emotion 16) and 80 subjects on a 128-detectors Somatom X.cite (Siemens Healthineers Erlangen, Germany). Images were acquired with low radiation dose parameters (110 kVps and ≤ 30 mAs) with CTDIvol 4.13–4.65 mGy.

The I-ELCAP [13] was used for diagnosis and follow-up of findings, with a retrospective analysis of the data collected in this cohort. They were staged by TNM, 8th edition [16].

### Image analysis

All CT images (540 examinations) were first reviewed by a highly experienced radiologist with 27 years’ experience. This radiologist identified the location and growth course of each nodule, blind to clinical and pathologic information. Subsequently, all identified lesions underwent segmentation using the open-source software ITK-SNAP [17] (version 3.8). The segmentation process was performed by an imaging expert and then carefully reviewed by a radiologist.

### Growth dynamics

To understand the growth patterns of lung nodules, we used the von Bertalanffy [18] (VB) growth equation,

$$\:\frac{\text{d}\text{V}}{\text{d}\text{t}}={\alpha\:}{\text{V}}^{{\beta\:}}.$$

**Fig. 1:**
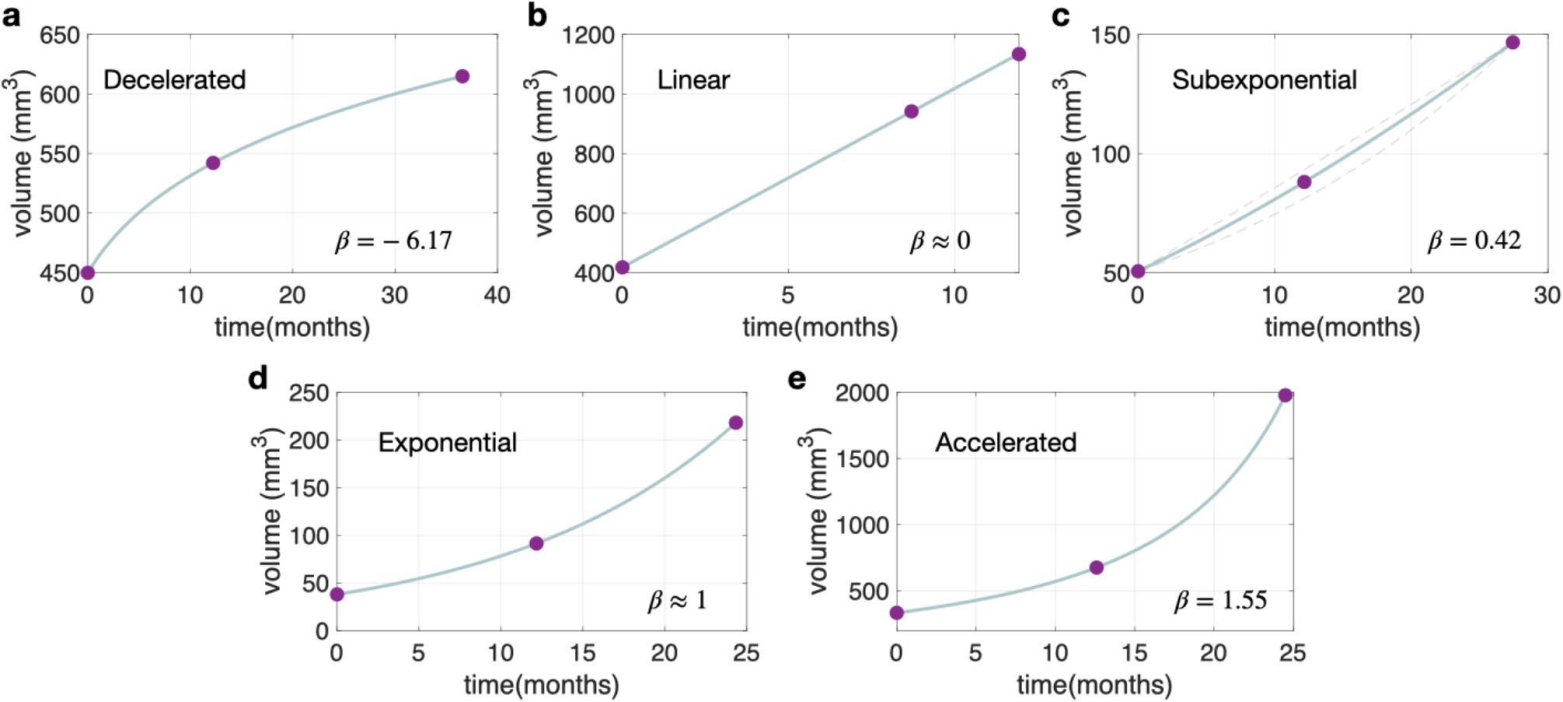
Growth dynamics of lung nodules according to growth exponent value. **a**. Decelerated growth of a hamartoma in a 60-year-old patient. **b**. Linear growth of an adenocarcinoma in a 70-year-old patient. **c** Subexponential growth of a hamartoma in a 55-year-old patient. **d**. Exponential growth of an adenocarcinoma in a 60-year-old patient. **e**. Accelerated growth of a small-cell lung cancer (SCLC) in a 69-year-old patient. Dots represent measured volumes, and the blue lines depict the outcome of the growth law for the fitted exponent

This equation has the advantage of categorizing various growth types based on the value of the growth exponent $\:{\beta\:}$. Specifically, it can identify decelerated ($\:{\beta\:}<0$), linear ($\:{\beta\:}=0$), subexponential ($\:{\beta\:}<1$), exponential $({\beta\:}=1),$ or accelerated ($\:{\beta\:}>1$) growth patterns. Examples of these five types of growth are shown in Fig. 1. To find the parameters for each patient, at least three longitudinal measurements ($\:{V}_{0},{V}_{1},{V}_{2}$), displaying an increase in volume over time, are required.

### Sensitivity analysis

Potential errors in tumor segmentation volumes could introduce variability into the estimated parameters, particularly the growth exponent ($\:\beta\:$). To ensure that minor fluctuations in computed volumes did not unduly influence the findings, a sensitivity analysis was conducted on the Von Bertalanffy model. Specifically, for each lesion, 200 simulations were run, adding random errors ranging from − 5% to + 5% to the volumetric data, and the resulting exponents computed. The robustness criterion in this case was to ensure that the median of the 200 computed $\:{\beta\:}^{*}$ values differed by less than 0.5 when compared to the value of $\:\beta\:$ obtained from the measured volumes, meaning $\:\left|{\beta\:}_{median}-\beta\:\right|<0.5$.

### Linear growth rate

The linear growth rate ($\:{\upnu\:}$) was computed as

$$\:{{\upnu\:}}_{\text{if}}=\frac{{V}_{f}-{V}_{i}}{{\Delta}t},$$

where $\:{V}_{i}$ represents the initial measured volume, $\:{V}_{f}$ a posterior measurement, and $\:{\Delta}t$ denotes the time interval between the two scans.

### Exponential growth rate

The exponential growth rate ($\:{\uplambda\:}$) was determined as

$$\:{{\uplambda\:}}_{\text{if}}=\frac{\text{log}\left({\text{V}}_{\text{f}}\right)-\text{log}\left({V}_{i}\right)}{{\Delta}t},$$

where, again, $\:{V}_{i}$ represents the initial volume, $\:{V}_{f}$ is a subsequent measurement, and $\:{\Delta}t$ represents the time interval between the two scans.

### Volume doubling time

Traditionally, the VDT in tumors is computed using the modified Schwartz Eqs. [19, 20], which assumes exponential growth based on the first two scans.

$$\:{\text{VDT}}_{\text{exp}}=\frac{\text{ln}2}{{{\uplambda\:}}_{01}}.$$

A VDT was also introduced, based on the tumor’s growth characteristics as defined by a scaling law. For each nodule, the parameters $\:{\alpha\:}$ and $\:{\beta\:}$ were calculated using the Von Bertalanffy equation, and the VDT computed using the formula

$$\:{\text{VDT}}_{{\beta\:}}=\frac{{\left(2{V}_{0}\right)}^{1-{\beta\:}}-{V}_{0}^{1-{\beta\:}}}{\left(1-{\beta\:}\right){\alpha\:}}.$$

### Statistical analysis

Statistical analyses were conducted using MATLAB 2022a (Mathworks) and SPSS (v.25) software. The normality of the variables was assessed by the Kolmogorov–Smirnov test. The Kruskal-Wallis test was employed with adjustments for multiple comparisons to ascertain statistically significant differences for non-parametric data. P-values smaller than 0.05 were considered to indicate a statistically significant difference.

To assess the effectiveness of the variables as classifiers, a receiver operating characteristic (ROC) curve was generated, and the area under the curve (AUC) was computed. The AUC serves as a measure of accuracy, providing insight into the performance of the variable in distinguishing between different groups.

Multivariate proportional hazard Cox analysis using the stepwise Wald method was employed to create predictive models. This approach assesses a group of variables and gradually eliminates the variable with the lowest statistical significance.

## Results

This study included a total of 180 participants, comprising 132 males and 48 females. The median age of the participants was 62 years (ranging from 51 to 81) with a dominance of adenocarcinoma histology (66.7%). Additional patient data are given in Table 1. Additional TNM data of the primaries on diagnosis are listed in Supplementary Table S1.

**Table 1:** Study participants characteristics. IQR: inter-quartile range, COPD: chronic obstructive pulmonary disease

	*n* (%)
Number of participants	180
Sex	
Male	132 (73.3)
Female	48 (26.7)
Age	
Median (IQR)	62 (59–66)
Smoking status	
Former smoker	80 (44.4)
Smoker	100 (55.6)
Packs per year	
Median (IQR)	30.6 (19–42)
COPD	
Normal	48 (26.7)
Mild	111 (61.7)
Moderate	16 (8.9)
Severe	5 (2.7)
Malignant tumors	**140 (77.8)**
Adenocarcinoma	120 (85.7)
Epidermoid carcinoma	11 (7.7)
Small-cell lung cancer (SCLC)	5 (3.6)
Large cell carcinoma	2 (1.4)
NSCLC-NOS	2 (1.4)
Benign nodules	**40 (22.2)**
Hamartoma	24 (60.0)
Granuloma	5 (12.5)
Carcinoid	2 (5.0)
Fibrosis	2 (5.0)
Sarcoidosis	1 (2.5)
Pneumonia	6 (15.0)
Stage	
I	112 (80.0)
II	12 (8.6)
III	15 (10.7)
IV	1 (0.7)
Classification (Malignant)	
Solid	81 (57.9)
Part-solid	28 (20.0)
Non-solid	31 (22.1)
Volume at first scan (mm^3^)	
Benign [Median (IQR)]	103.5 (49.5–414.3)
Malignant [Median (IQR)]	179.3 (64.3–527.2)

The median time interval between first and second scans was 12.2 months (IQR: 6.2–13.9), while between the first and third scans was 24.3 months (IQR: 14.37–30.8).

### Growth dynamics

**Fig. 2:**
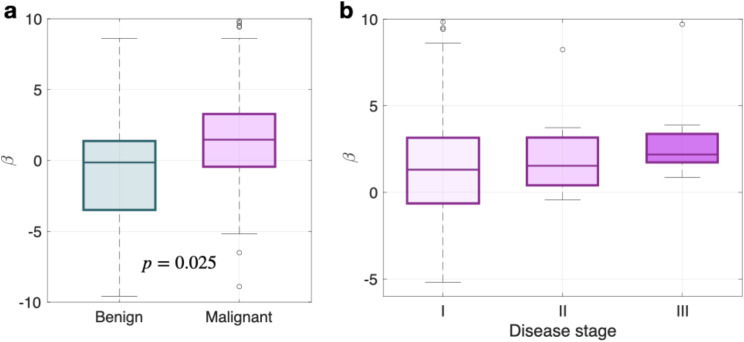
Box plots for the growth exponents of lung nodules according to (**a**) malignancy (benign, *n* = 40; malignant, *n* = 140) and (**b**) TNM stage (stage I, *n* = 112; stage II, *n* = 12; stage III, *n* = 15). An additional malignant nodule, not included in the figure, presented stage IV ($\:\beta\:$=0.77). P-value corresponds to the Kruskal-Wallis test

Among the benign lesions analyzed, half (20) displayed decelerated growth, as evidenced by growth exponents smaller than 0. For eight lesions (20.0%), the growth pattern was subexponential ($\:0<{\beta\:}<1$), one of them grew exponentially, while the remaining lesions (11) exhibited accelerated behavior. In contrast, for malignant nodules, one displayed linear growth ($\:{\beta\:}=0$), and six followed exponential growth patterns ($\:{\beta\:}=1$). However, most of the malignant nodules (83 out of 140) showed accelerated growth. The remaining malignant nodules showed either subexponential (13) or decelerated growth (37). See Fig. 2.a, Table 2, and Supplementary Figure S1 for more details. Although growth exponents were also categorized based on the TNM staging (Fig. 2.b), there was no statistical significance in the dataset.

**Table 2:** Types of growth according to the value of the growth exponent $\:\varvec{\beta\:}$ for benign (*n* = 40) and malignant (*n* = 140) lung nodules

Type of growth	$\:\varvec{\beta\:}$	Benign	Malignant
Decelerated	$\:\le\:-0.1$	20	37
Linear	$\:-0.1<\beta\:\le\:0.1$	0	1
Subexponential	$\:0.1<\beta\:\le\:0.9$	8	13
Exponential	$\:0.9<\beta\:\le\:1.1$	1	6
Accelerated	$\:>1.1$	11	83

A sensitivity analysis, detailed in the “Methods-Sensitivity analysis” section, revealed that certain lesions’ growth exponents were susceptible to variations of up to ± 5% in volumes. However, a substantial portion of the dataset, 76.6% (*n* = 138), exhibited robustness to these volume fluctuations when computing the exponent $\:\beta\:$ and 42 showed higher variability and were excluded from this analysis. This demonstrated the reliability and consistency of our findings across these different approaches. Tables S2 and S3 describe the types of growth after either removing the smallest lesions (< 100 mm^3^) (Table S2) or performing the sensitivity analysis (Table S3), respectively.

When classifying lung cancers into solid, part-solid and non-solid nodules, it was observed that non-solid nodules grew slower than exponential and growth differences with solid and part-solid tumors were statistically significant (Figure S2.a). However, solid and part-solid nodules did not show significant differences in their growth dynamics.

The values for VDT in the case of malignant tumors fall within a similar range (12.4–19.9 months), demonstrating no significant differences across different disease stages or when accounting for the different histology (Figure S3).

### Benign and malignant lung lesions classification

Although malignant nodules were larger at baseline (median 171 mm^3^) than benign ones (median 84 mm^3^), with no statistical significance, *p* = 0.08 (Figure S4), at subsequent scans the differences did become statistically significant: *p* = 0.007 at t_1_ (255 mm^3^ vs. 119 mm^3^) and *p* < 0.001 at t_2_ (519 mm^3^ vs. 155 mm^3^).

When examining the attenuation (Hounsfield Unit – HU) within the structure, benign nodules exhibited longitudinal stability (median values − 146, -156 and − 131, at each time point), while malignant nodules showed progressive increase (median − 314, -291, -233). Lesion classification using HU at the first scan achieved a ROC-AUC of 0.758 with sensitivity and specificity of 0.750 and 0.700, respectively (*p* < 0.001). See Figure S5 for further details.

Lesion mass, computed as the product of the lesion volume and attenuation differentiated benign from malignant lesions statistically at each time point (*p* < 0.001) (Figure S6a). At baseline, the ROC-AUC classifier was 0.689 (0.625 sensitivity, 0.701 specificity). Their β were useless for distinguishing growth patterns (Figure S6a.b-c).

All volume-based approximations for discriminating between benign and malignant nodules showed statistically significant differences (*p* < 0.001), with ROC curve analyses resulting in AUC values ranging from 0.634 to 0.871. A comprehensive summary of the methods used is shown in Table 3.

**Table 3:** Summary of the discriminatory power of all the methods explored in this study. AUC – area under the curve of the receiver operating characteristic (ROC) curve, and their corresponding sensitivity and specificity values

Approximation	Time-points	AUC	Sensitivity	Specificity
VB equation	$\:\beta\:$ ($\:{V}_{0},{V}_{1},{V}_{2}$)	0.671	0.700	0.650
	$\:\alpha\:$ ($\:{V}_{0},{V}_{1},{V}_{2}$)	0.634	0.600	0.688
	VDT ($\:{V}_{0},{V}_{1},{V}_{2}$)	0.771	0.750	0.796
Linear	$\:{V}_{0},{V}_{1}$	0.769	0.786	0.650
	$\:{V}_{0},{V}_{2}$	**0.871**	**0.843**	**0.775**
	$\:{V}_{1},{V}_{2}$	0.856	0.807	0.800
	$\:{V}_{0},{V}_{1},{V}_{2}$	0.866	0.807	0.800
Exponential	$\:{V}_{0},{V}_{1}$	0.733	0.693	0.775
	$\:{V}_{0},{V}_{2}$	0.831	0.821	0.800
	$\:{V}_{1},{V}_{2}$	0.810	0.771	0.775
	$\:{V}_{0},{V}_{1},{V}_{2}$	0.842	0.814	0.800
	VDT ($\:{V}_{0},{V}_{1}$)	0.708	0.750	0.693
	VDT ($\:{V}_{0},{V}_{1},{V}_{2}$)	0.817	0.775	0.814
Volume	$\:{V}_{0}$	0.591	0.607	0.625
Attenuation	$\:{\text{HU}}_{0}$	0.758	0.750	0.700
Mass	$\:{\text{V}}_{0}\cdot\:{\text{HU}}_{0}$	0.689	0.625	0.707

**Fig. 3:**
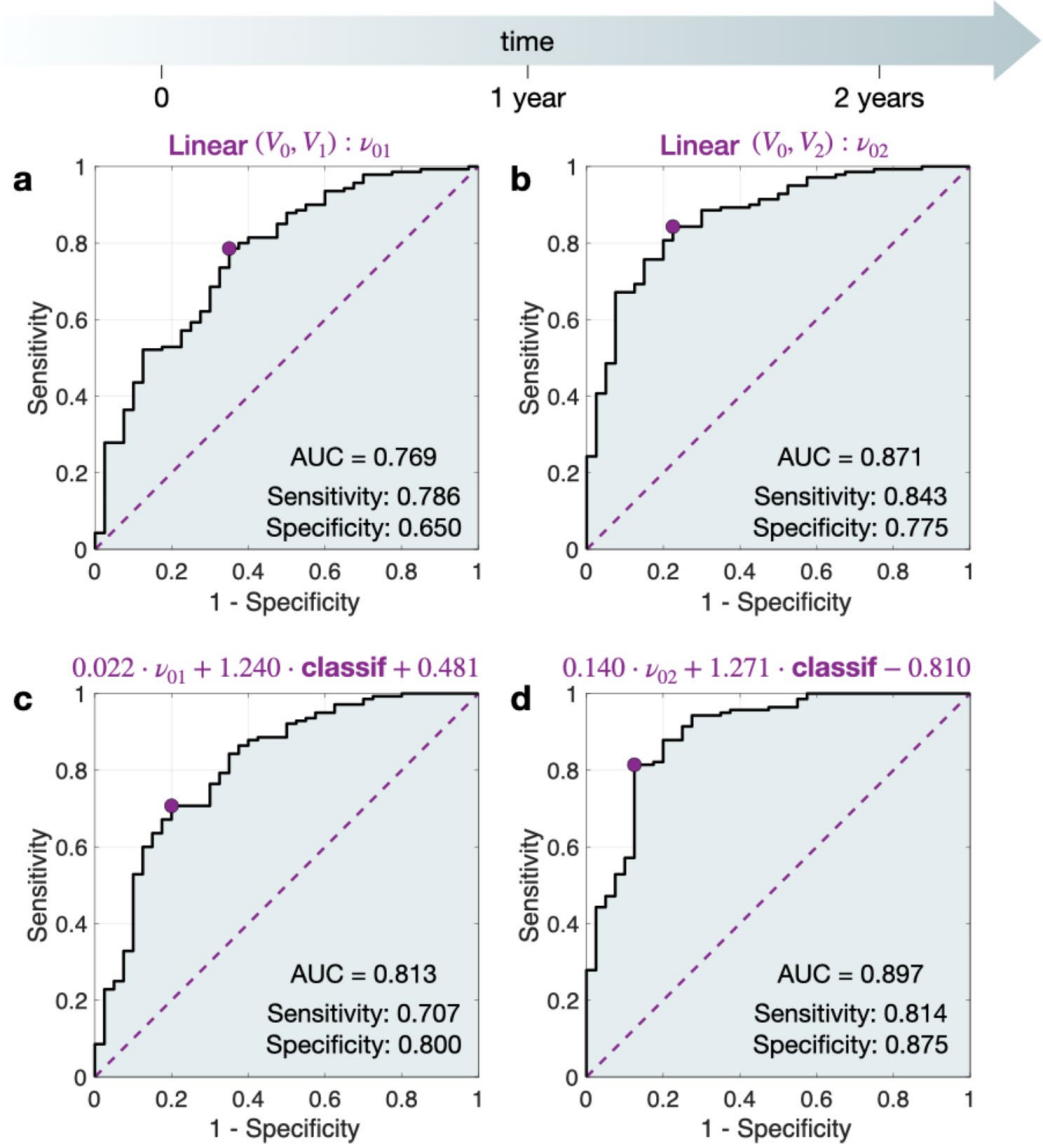
Receiver operating characteristic (ROC) curves for the best classifiers distinguishing between benign and malignant lung nodules. **a**. Linear model with measurements from the first and second scans, $\:{\nu\:}_{01}$. **b**. Linear model with measurements from the first and third scans, $\:{\nu\:}_{02}$. (c) Linear model (first and second scans, $\:{\nu\:}_{01}$) combined with the classification of the nodule as solid, part-solid or non-solid. (d) Linear model (first and third scans, $\:{\nu\:}_{02}$) combined with the nodule classification. AUC – Area under the curve. HU – Hounsfield Unit

The linear model using measurements from the first and third points ($\:{V}_{0},{V}_{2}$) resulted in the best ROC-AUC, achieving a value of 0.871, with a sensitivity of 0.843 and specificity of 0.775 (Fig. 3.b). However, employing the linear model between the first and second scans ($\:{V}_{0},{V}_{1}$) achieved a ROC-AUC of 0.769, with 0.786 sensitivity and 0.650 specificity, effectively providing classification one year in advance (Fig. 3.a).

Exploring various multivariable approximations (Supplementary Table S4), the optimal classification emerged by combining the linear model (first and third scans) with the nodule classification as solid, part-solid or non-solid, achieving a ROC-AUC of 0.897 (sensitivity 0.814, specificity 0.875) (Fig. 3.d). The linear approximation with the first two scans alongside the mean image yielded a ROC-AUC of 0.813 (sensitivity 0.707, specificity 0.800) (Fig. 3.c).

## Discussion

Growth volume in lung nodules from a I-ELCAP screening program showed that most primary lung cancers showed an accelerated growth rate. Of the various models, the linear growth rate, computed using the first and third points, was the most discriminative (ROC-AUC = 0.871).

Several studies have suggested that the growth dynamics of lung tumors follows an exponential law. In one study, 47 lesions were examined, revealing that the exponential function was a suitable fit for most cases [7]. However, on analyzing the dynamics of three specific lesions in their paper (see Figure S7), none was best fitted by an exponential function ($\:{\beta\:}=1$). Similarly, a study of 60 non-screening-detected lung cancers emphasized the accuracy of the exponential growth description, but acknowledged variations in larger, fast-growing nodules [8]. Despite identifying adenocarcinomas as best represented by exponential growth, another study found substantial heterogeneity in growth patterns, as illustrated in their Fig. 9 (see Suppl. Figure S8).

An ancillary study, challenging earlier assumptions of exponential growth, found that lung cancers lacked consistent patterns of linear, exponential, or Gompertzian growth [11]. However, their study did not find significant variations when comparing these growth patterns with those observed in benign nodules.

The largest lung cancer screening study, NELSON [21], included 7557 participants who underwent CD screening at baseline (first round), 1 year later if inconclusive (second round), and 3 years later. As in our study, most malignancies were adenocarcinomas. However, the fact that we imposed a strict inclusion criterion, namely that three follow-ups were required and that lesions should grow continuously, resulted in the exclusion of a significant number of cases from our study, benign if no growth, but also more aggressive tumors, as patients diagnosed at the first or second follow-up were excluded. Most likely, the cases in our study should be compared to those in NELSON, who had indeterminate results (19.2%) in the second round.

When assessing nodules based on their components, non-solid nodules exhibited slower growth in this study, consistent with previous findings [9, 22]. Excluding non-solid lesions improved the classification between benign and malignant lesions, enhancing the AUC-ROC from 0.671 to 0.705. The classification further improved to an AUC-ROC of 0.722 when considering only solid nodules. Lung nodule size, along with other variables like nodule location and the presence of distant tumors, plays a crucial role in stage classification [16]. In the dataset, malignant tended to be larger than benign lesions, albeit not in a statistically significant manner at baseline.

For the classification between benign and malignant nodules, previous studies have examined the mean VDT in CT-based screening studies. A substantial difference in VDT was found to be a valuable indicator. In line with this, Xu et al. [23]. proposed that a VDT of less than 400 days could serve as a predictor for malignancy, and they obtained a positive predictive value of 63%. However, although Hammer et al. [24] also noted that malignant nodules generally exhibited faster growth compared to benign nodules, they found a substantial overlap, rendering VDT unsuitable for reliable classification. In our cohort, using the VDT yielded worse results compared to other methods.

Remarkably, mean VDT observed for NSCLC varied depending on the study setting [25]. For symptomatic patients in routine medical care, the mean VDT was approximately 135 days [26–29]. In contrast, in CT screening studies, the mean VDT extended to around 480 days [20, 30–32]. This marked difference is consistent with the accelerated growth pattern previously reported [12]. In that framework, one would expect that in screening studies, where tumors are caught in their initial stages of development, they will be observed to grow more slowly than when they are symptomatic thus leading to different perceived aggressiveness patterns.

Though linear approximation with only two points from two-year interval was the best classifier, was further enhanced when combined with the CT morphology. The resulting AUC values were 0.871 and 0.897, respectively. The latter points to a very high classification accuracy that can easily be achieved in clinical practice. Notably, this classification can be anticipated to the first year, second scan, although with a reduction in efficacy. The linear approximation alone yielded an AUC of 0.769, which increased to 0.813 when combined with the lesion classification. Previous studies had achieved comparable classification results by using radiomic studies with over 1000 features [33, 34]. The classification method used in this study stands out for its simplicity, relying on the straightforward volumetric growth between two time points.

Our study has different strengths. Firstly, our annual interval scans provide a unique approach, addressing the lack of consensus on the optimal frequency for lung nodule screening and management, as no uniform approach or guideline exist [35]. Lung-RADS definition of growth, for example, does not address situations in which the interval between two assessments exceeds 12 months [36]. Secondly, the robust volume measurements align with updated guidelines, such as Lung-RADS 1.1, reflecting a significant advancement supported by studies like NELSON, which demonstrated substantial benefits with volumetric measurements among the largest LCS studies [37].

The fact that manual segmentations were performed by a single expert could be perceived as a limitation of the study. However, all segmentations were validated by a senior radiologist with more than 20 years of experience. It is worth pointing out that interobserver variability in manual segmentation may not significantly influence the ability to extract accurate radiomic features for lung tumors on CT [38]. In the case of semi-automated volumetry, interobserver correlation is high [39] –Spearman *r* = 0.99 -. In fact, compared to diameter measurements volumetry achieves a significantly smaller interobserver variance and advanced volumetry algorithms tend to be independent of observer experience [40].

The study’s limitations stem mainly from its retrospective design. Solid components within part-solid lesions and the development of solid nodules from non-solid nodules at baseline were not measured, limiting insights into the invasive component [9]. Additionally, the inherent prevalence of TNM stage I in a cancer screening program and the dominance of adenocarcinoma hindered meaningful histological comparisons.

In conclusion, the study improves understanding of the growth dynamics of lung nodules, challenging established assumptions. A generalized accelerated growth pattern in malignant lung tumor dynamics has been shown to exist. Moreover, linear growth rate practicality obtained from distant time points is highlighted as a reliable classifier of lung nodule malignancy, together with CT morphology (solid, part-solid or non-solid). This straightforward approach holds promise for informing and enhancing screening and diagnostic lung cancer methods.

### Electronic supplementary material

Below is the link to the electronic supplementary material.

Supplementary Material 1

## Data Availability

The datasets used and/or analyzed during the current study are available from the corresponding author on reasonable request.

## Abbreviations

I-ELCAP: International Early Lung Cancer Action Program
LCS: Lung cancer screening
NSCLC: Non-small-cell lung cancer
VDT: Volume doubling time

## References

[1] Henschke CI, Yankelevitz DF, Yip R, et al. Lung cancers diagnosed at annual CT screening: volume doubling times. Radiology. 2012;263(2):578–83. 10.1148/radiol.12102489. 22454506 10.1148/radiol.12102489 PMC3329268

[2] Siegel RL, Miller KD, Wagle NS, Jemal A. Cancer statistics, 2023. CA Cancer J Clin. 2023;73(1):17–48. 10.3322/caac.21763. 36633525 10.3322/caac.21763

[3] Muthusamy B, Patil PD, Pennell NA. Perioperative systemic therapy for Resectable non–small cell Lung Cancer. JNCCN J Natl Compr Cancer Netw. 2022;20(8):953–61. 10.6004/jnccn.2022.7021. 10.6004/jnccn.2022.7021 35948038

[4] Rami-Porta R, Call S, Dooms C, et al. Lung cancer staging: a concise update. Eur Respir J. 2018;51(5). 10.1183/13993003.00190-2018. 10.1183/13993003.00190-2018 29700105

[5] Jones GS, Baldwin DR. Recent advances in the management of lung cancer. Clin Med (Lond). 2018;18(Suppl 2):s41–6. 10.7861/clinmedicine.18-2-s41. 29700092 10.7861/clinmedicine.18-2-s41 PMC6334032

[6] MacMahon H, Naidich DP, Goo JM, et al. Guidelines for management of incidental pulmonary nodules detected on CT images: from the Fleischner Society 2017. Radiology. 2017;284(1):228–43. 10.1148/radiol.2017161659. 28240562 10.1148/radiol.2017161659

[7] Heuvelmans MA, Vliegenthart R, de Koning HJ, et al. Quantification of growth patterns of screen-detected lung cancers: the NELSON study. Lung Cancer. 2017;108:48–54. 10.1016/j.lungcan.2017.02.021. 28625647 10.1016/j.lungcan.2017.02.021

[8] Mets OM, Chung K, Zanen P, et al. In vivo growth of 60 non-screening detected lung cancers: a computed tomography study. Eur Respir J. 2018;51(4). 10.1183/13993003.02183-2017. 10.1183/13993003.02183-2017 29650547

[9] de Margerie-Mellon C, Ngo LH, Gill RR, et al. The growth rate of subsolid lung adenocarcinoma nodules at chest CT. Radiology. 2020;297(1):189–98. 10.1148/radiol.2020192322. 32749206 10.1148/radiol.2020192322

[10] Ko JP, Berman EJ, Kaur M, et al. Pulmonary nodules: growth rate assessment in patients by using serial CT and three-dimensional volumetry. Radiology. 2012;262(2):662–71. 10.1148/radiol.11100878. 22156993 10.1148/radiol.11100878 PMC3267080

[11] Lindell RM, Hartman TE, Swensen SJ, Jett JR, Midthun DE, Mandrekar JN. 5-Year lung cancer screening experience: growth curves of 18 lung cancers compared to histologic type, CT attenuation, stage, survival, and size. Chest. 2009;136(6):1586–95. 10.1378/chest.09-0915. 19581354 10.1378/chest.09-0915 PMC2789925

[12] Pérez-García VM, Calvo GF, Bosque JJ, et al. Universal scaling laws rule explosive growth in human cancers. Nat Phys. 2020;16(12):1232–7. 10.1038/s41567-020-0978-6. 33329756 10.1038/s41567-020-0978-6 PMC7116451

[13] International Early Lung Cancer Action Program Investigators. International Early Lung Cancer Action Program protocol. Accessed September 23. 2023. www.IELCAP.org/protocols.

[14] Cervera Deval J, Barrios Benito M, Peñalver Cuesta JC, et al. Lung Cancer Screening: Survival in an extensive early detection program in Spain (I-ELCAP). Arch Bronconeumol. 2022;58(5):406–11. 10.1016/j.arbres.2021.10.005. 35312494 10.1016/j.arbres.2021.10.005

[15] Patel AR, Patel AR, Singh S, Singh S, Khawaja I. Global Initiative for Chronic Obstructive Lung Disease: the changes made. Cureus Published Online June. 2019;24. 10.7759/cureus.4985. 10.7759/cureus.4985 PMC6701900 31453045

[16] Detterbeck FC, Boffa DJ, Kim AW, Tanoue LT. The Eighth Edition Lung Cancer Stage classification. Chest. 2017;151(1):193–203. 10.1016/j.chest.2016.10.010. 27780786 10.1016/j.chest.2016.10.010

[17] Yushkevich P, Piven J, Hazlett HC, et al. User-guided 3D active contour segmentation of anatomical structures: significantly improved efficiency and reliability. NeuroImage. 2006;31(3):1116–28. 16545965 10.1016/j.neuroimage.2006.01.015

[18] von Bertalanffy L. Quantitative laws in metabolism and growth. Q Rev Biol. 1957;32(3):217–31. 13485376 10.1086/401873

[19] Schwartz M. A biomathematical approach to clinical tumor growth. Cancer. 1961;14:1272–94. 13909709 10.1002/1097-0142(196111/12)14:6<1272::AID-CNCR2820140618>3.0.CO;2-H

[20] Hasegawa M, Sone S, Takashima S, et al. Growth rate of small lung cancers detected on mass CT screening. Br J Radiol. 2000;73(876):1252–9. 11205667 10.1259/bjr.73.876.11205667

[21] van Klaveren RJ, Oudkerk M, Prokop M, et al. Management of lung nodules detected by volume CT scanning. N Engl J Med. 2009;361(23):2221–9. 10.1056/NEJMoa0906085. 19955524 10.1056/NEJMoa0906085

[22] Yankelevitz DF, Yip R, Smith JP, et al. CT screening for lung cancer: nonsolid nodules in baseline and annual repeat rounds. Radiology. 2015;277(2):555–64. 10.1148/radiol.2015142554. 26101879 10.1148/radiol.2015142554 PMC4627436

[23] Xu DM, Van Derzaag-Loonen HJ, Oudkerk M, et al. Smooth or attached solid indeterminate nodules detected at baseline CT screening in the NELSON study: Cancer risk during 1 year of follow-up. Radiology. 2009;250(1):264–72. 10.1148/radiol.2493070847. 18984780 10.1148/radiol.2493070847

[24] Hammer MM, Gupta S, Byrne SC. Volume Doubling Times of Benign and Malignant Nodules in Lung Cancer Screening. *Curr Probl Diagn Radiol*. Published online 2023. 10.1067/j.cpradiol.2023.06.014 10.1067/j.cpradiol.2023.06.014 PMC10592400 37451949

[25] Detterbeck FC, Gibson CJ. Turning Gray: the natural history of Lung Cancer over Time. J Thorac Oncol. 2008;3(7):781–92. 18594326 10.1097/JTO.0b013e31817c9230

[26] Geddes DM. The natural history of lung cancer: a review based on rates of tumour growth. Br J Dis Chest. 1979;73:1–17. 435370 10.1016/0007-0971(79)90002-0

[27] Mizuno T, Masaoka A, Shibata K, Tanaka H, Niwa H. Comparison of actual Survivorship after Treatment with Survivorship predicted by actual tumor-volume Doubling Time from Tumor Diameter at First Observation. Cancer. 1984;53(12):2716–20. 6326992 10.1002/1097-0142(19840615)53:12<2716::AID-CNCR2820531227>3.0.CO;2-N

[28] Usuda K, Saito Y, Sagawa M, et al. Tumor doubling time and prognostic assessment of patients with primary lung cancer. Cancer. 1994;74(8):2239–44. 7922975 10.1002/1097-0142(19941015)74:8<2239::AID-CNCR2820740806>3.0.CO;2-P

[29] Jennings SG, Winer-Muram HT, Tann M, Ying J, Dowdeswell I. Distribution of stage I lung cancer growth rates determined with serial volumetric CT measurements. Radiology. 2006;241(2):554–63. 10.1148/radiol.2412051185. 17005771 10.1148/radiol.2412051185

[30] Takashima S, Maruyama Y, Hasegawa M, et al. CT findings and progression of small peripheral lung neoplasms having a replacement growth pattern. Am J Roentgenol. 2003;180(3):817–26. www.ajronline.org. 12591704 10.2214/ajr.180.3.1800817

[31] Sone S, Nakayama T, Honda T, et al. Long-term follow-up study of a population-based 1996–1998 mass screening programme for lung cancer using mobile low-dose spiral computed tomography. Lung Cancer. 2007;58(3):329–41. 10.1016/j.lungcan.2007.06.022. 17675180 10.1016/j.lungcan.2007.06.022

[32] Lindell RM, Hartman TE, Swensen SJ, et al. Five-year lung cancer screening experience: CT appearance, growth rate, location, and histologic features of 61 lung cancers. Radiology. 2007;242(2):555–62. 10.1148/radiol.2422052090. 17255425 10.1148/radiol.2422052090

[33] Huang P, Park S, Yan R, et al. Added value of computer-aided CT image features for early lung cancer diagnosis with small pulmonary nodules: a matched case-control study. Radiology. 2018;286(1):286–95. 10.1148/radiol.2017162725. 28872442 10.1148/radiol.2017162725 PMC5779085

[34] Paez R, Kammer MN, Balar A, et al. Longitudinal lung cancer prediction convolutional neural network model improves the classification of indeterminate pulmonary nodules. Sci Rep. 2023;13(1). 10.1038/s41598-023-33098-y. 10.1038/s41598-023-33098-y PMC10105767 37061539

[35] Bonney A, Malouf R, Marchal C, et al. Impact of low-dose computed tomography (LDCT) screening on lung cancer-related mortality. Cochrane Database Syst Reviews. 2022;2022(8). 10.1002/14651858.CD013829.pub2. 10.1002/14651858.CD013829.pub2 PMC9347663 35921047

[36] Chelala L, Hossain R, Kazerooni EA, Christensen JD, Dyer DS, White CS. Lung-RADS version 1.1: challenges and a look ahead, from the AJR special series on radiology reporting and data systems. Am J Roentgenol. 2021;216(6):1411–22. 10.2214/AJR.20.24807. 33470834 10.2214/AJR.20.24807

[37] Zhao YR, Xie X, De Koning HJ, Mali WP, Vliegenthart R, Oudkerk M. NELSON lung cancer screening study. Cancer Imaging. 2011;11(SPEC ISS A). 10.1102/1470-7330.2011.9020. 10.1102/1470-7330.2011.9020 PMC3266562 22185865

[38] Hershman M, Yousefi B, Serletti L, et al. Impact of Interobserver Variability in Manual Segmentation of Non-small Cell Lung Cancer (NSCLC) applying low-rank radiomic representation on computed tomography. Cancers (Basel). 2021;13(23):5985. 10.3390/cancers13235985. 34885094 10.3390/cancers13235985 PMC8657389

[39] Gietema HA, Wang Y, Xu D, et al. Pulmonary nodules detected at Lung Cancer Screening: Interobserver variability of Semiautomated volume measurements. Radiology. 2006;241(1):251–7. 10.1148/radiol.2411050860. 16908677 10.1148/radiol.2411050860

[40] Bolte H, Jahnke T, Schäfer FKW, et al. Interobserver-variability of lung nodule volumetry considering different segmentation algorithms and observer training levels. Eur J Radiol. 2007;64(2):285–95. 10.1016/j.ejrad.2007.02.031. 17433595 10.1016/j.ejrad.2007.02.031

