# Supplementary material for: Growth dynamics of lung nodules: implications for classification in lung cancer screening
Source: Cancer Imaging. 2024 Aug 26;24:113. doi: 10.1186/s40644-024-00755-y (PMC11346294; doi:10.1186/s40644-024-00755-y)
Supplement: Supplementary file 1 — Supplementary Material 1 [file 40644_2024_755_MOESM1_ESM.pdf]

Supplementary Material for: “Growth Dynamics of Lung Nodules: Implications for Classification in lung cancer screening “

Beatriz Ocaña-Tienda, Alba Eroles-Simó, Julián Pérez-Beteta, Estanislao Arana, Víctor M. Pérez-García

TNM	n (%)
**T1miN0M0**	7
T1aN0M0	71
T1aN0M1b	1
T1aN1M0	2
T1aN2M0	4
T1aNXM0	1
T1bN0M0	15
T1bN2M0	1
T1bN3M0	1
T1cN0M0	3
T1cN1M0	1
T1cN2M0	2
T1N0M0	3
T1N2M0	1
T1N3M0	1
T2aN0M0	11
T2aN1M0	1
T2aN2M0	3
T2bN0M0	1
T3N0M0	6
T3N1M0	1
T4N0M0	3

Table S1. TNM staging of the patients included in the study.

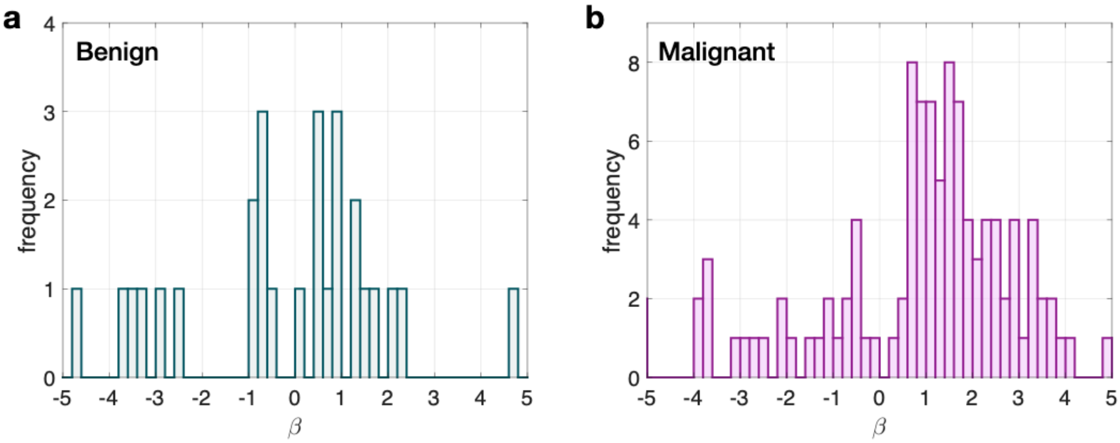

***Figure S1. Distribution of the growth exponents β** for a. benign lung nodules (n=40) and b. malignant lung lesions (n=140). Some outliers (with high/small values of the growth exponent) are out of the representation limits.*

Type of growth	β	Benign	Malignant
Decelerated	≤ -0.1	13	29
Linear	$-0.1 < \beta \leq 0.1$	0	1
Between linear and exponential	$0.1 < \beta \leq 0.9$	0	2
Exponential	$0.9 < \beta \leq 1.1$	1	2
Accelerated or over-exponential	> 1.1	5	56

Table S2. Types of growth according to the value of the growth exponent β for benign (n=19) and malignant (n=90) lung nodules after removing the smallest lesions (< 100 mm³).

Type of growth	β	Benign	Malignant
Decelerated	≤ -0.1	14	23
Linear	$-0.1 < \beta \leq 0.1$	0	1
Between linear and exponential	$0.1 < \beta \leq 0.9$	6	11
Exponential	$0.9 < \beta \leq 1.1$	1	5
Accelerated or over-exponential	> 1.1	9	68

Table S3. Types of growth according to the growth exponent β for benign (n=30) and malignant (n=108) lung nodules after performing a sensitivity analysis.

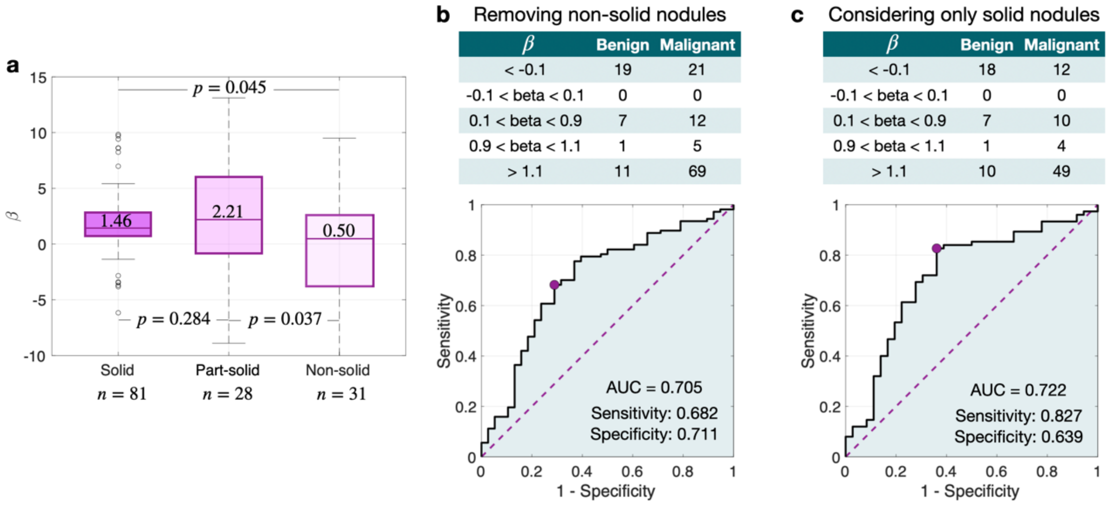

b Removing non-solid nodules

β	Benign	Malignant
< -0.1	19	21
-0.1 < beta < 0.1	0	0
0.1 < beta < 0.9	7	12
0.9 < beta < 1.1	1	5
> 1.1	11	69

c Considering only solid nodules

β	Benign	Malignant
< -0.1	18	12
-0.1 < beta < 0.1	0	0
0.1 < beta < 0.9	7	10
0.9 < beta < 1.1	1	4
> 1.1	10	49

Figure S2. Growth dynamics of lung nodules according to its classification: solid, part-solid and non-solid. a. *Growth exponent β for each group. P values correspond to the Kruskal-Wallis test.* ***b****. Types of growth attending to the value of the growth exponent and ROC curve for the classification between benign and malignant nodules for solid and part-solid lesions (n=145).* ***c****. Types of growth attending to the value of the growth exponent and ROC curve for the classification between benign and malignant nodules for solid lesions (n=111).*

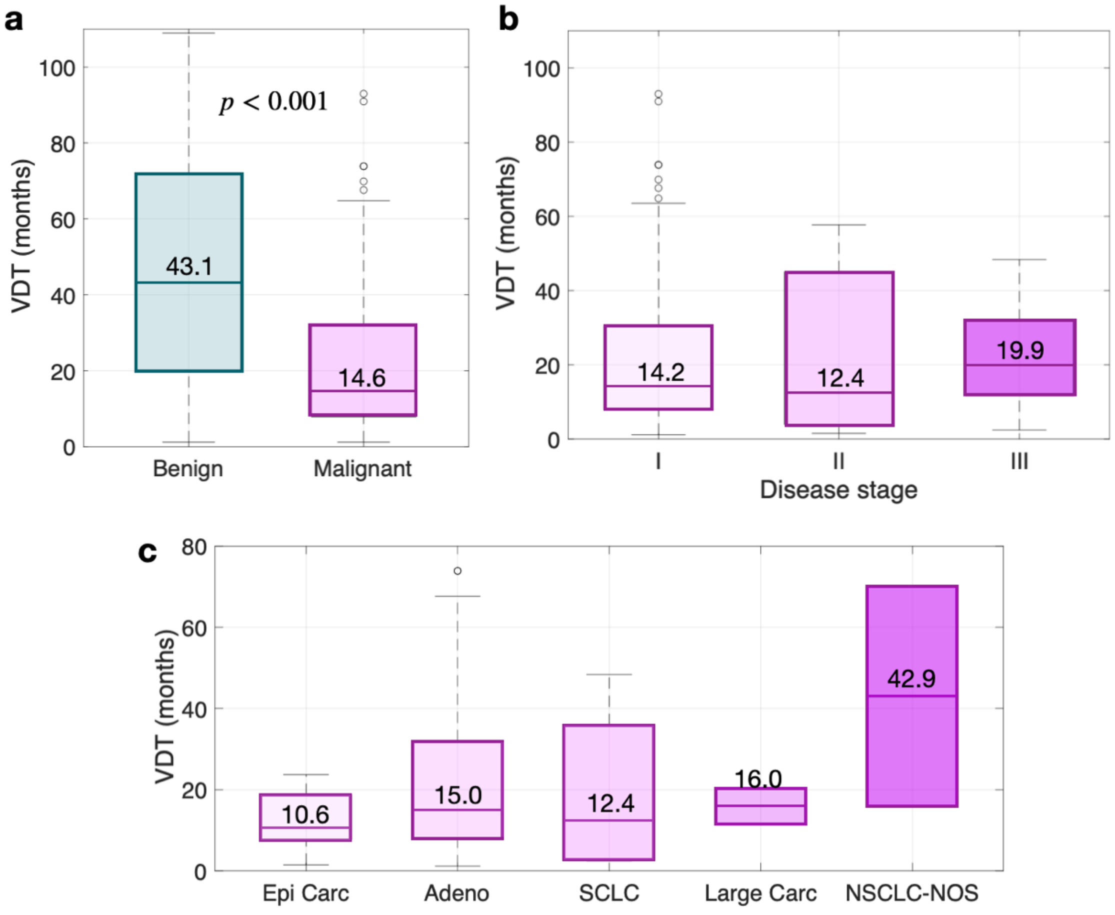

Figure S3. Box plots for the Volume doubling time (VDT) for different subgroups. a*. Benign vs malignant. The p-value corresponds to the Kruskal-Wallis test.* ***b****. According to disease stage.* ***c.*** *Primary subtype. Values inside the boxes correspond to the median values.*

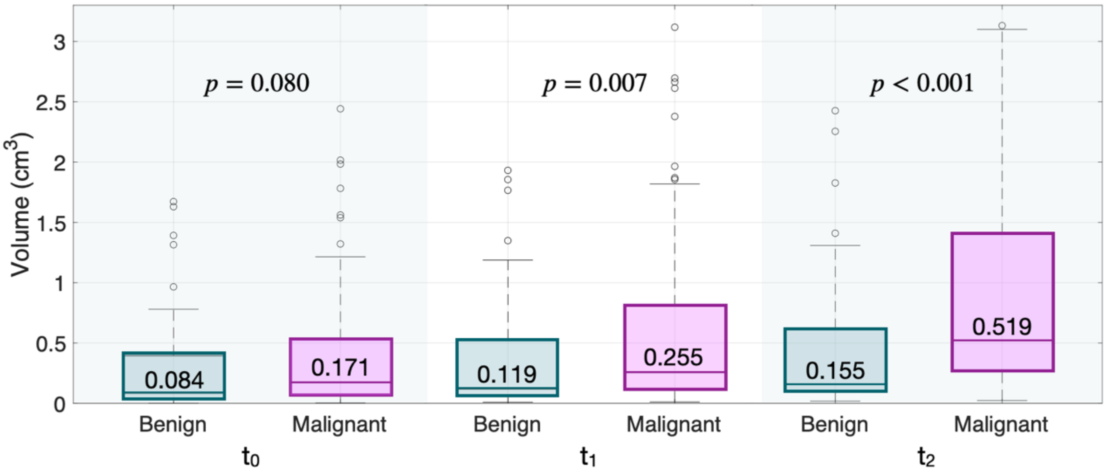

Figure S4. Total volume comparison of benign (n=40) and malignant (n=140) lung nodules at three measured time points*. P-values denote Kruskal-Wallis test results.*

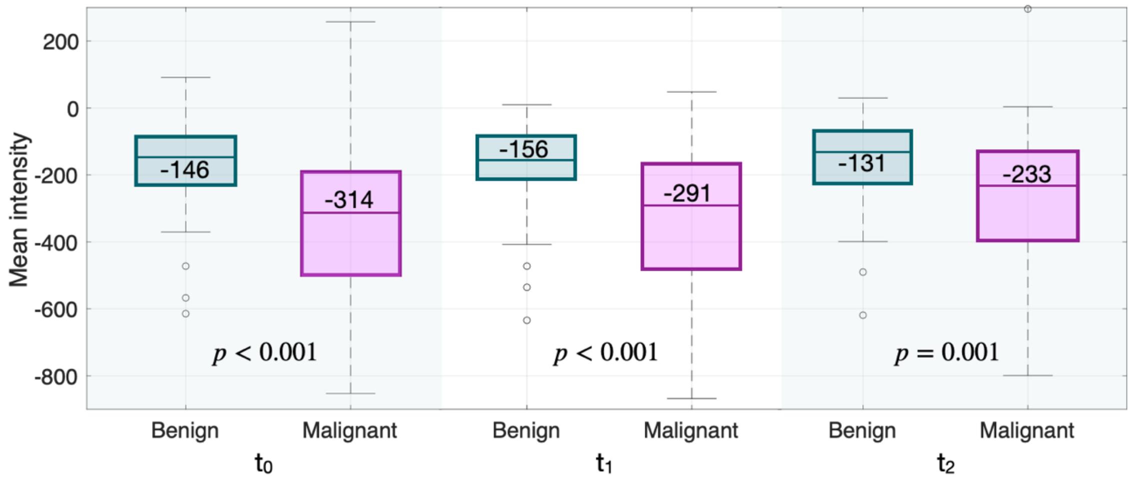

Figure S5. Image attenuation comparison of benign (n=40) and malignant (n=140) lung nodules at three measured time points*. P-values denote Kruskal-Wallis test results.*

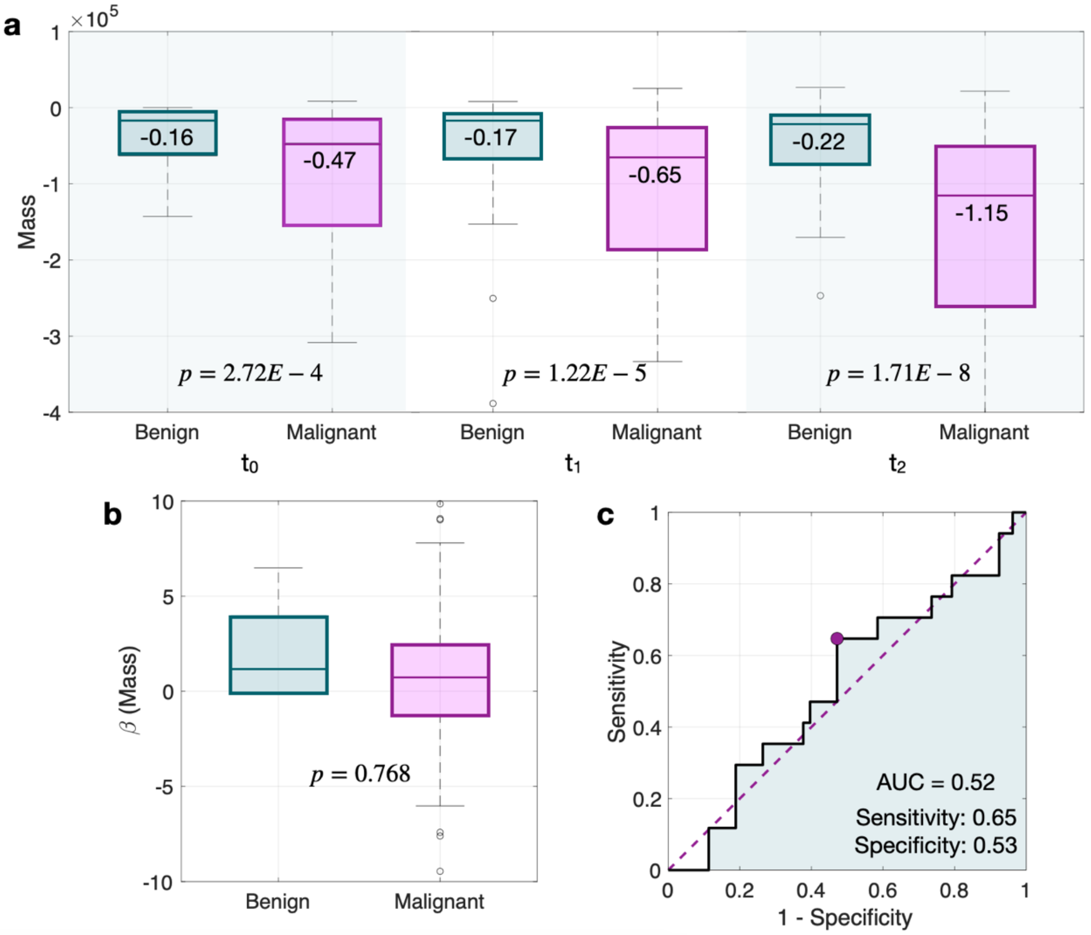

Figure S6. Mass comparison between benign (n=40) and malignant (n=140) lung nodules. a. *At the three measured time points.* ***b.*** *Box plots for the growth exponents β computed from the mass.* ***c.*** *Receiver operating characteristic (ROC) curve for β from mass to classify between benign and malignant lung nodules. P-values denote Kruskal-Wallis test results.*

Variables included	AUC	Sensitivity	Specificity
Linear (V_0, V_2) + VDT (β)	0.8715	0.847	0.775
Linear (V_0, V_2) + β	0.8705	0.843	0.775
Linear (V_0, V_2) + V_0	0.8725	0.757	0.875
Linear (V_0, V_2) + Linear (V_0, V_1)	0.8748	0.829	0.800
Linear (V_0, V_2) + Linear (V_0, V_1) + V_0	0.8732	0.750	0.875
Linear (V_0, V_2) + Linear (V_0, V_1) + VDT (β)	0.8785	0.825	0.800
Linear (V_0, V_2) + VDT $(\beta))$ + V_0	0.8876	0.825	0.825
Linear (V_0, V_2) + Linear (V_0, V_1) + β	0.8757	0.829	0.800
Linear (V_0, V_2) + β + V_0	0.8720	0.757	0.875
Linear (V_0, V_2) + attenuation	0.8913	0.879	0.850
Linear (V_0, V_1) + attenuation	0.8048	0.836	0.725
Linear (V_0, V_2) + solid classification	**0.8971**	0.814	0.875
Linear (V_0, V_1) + solid classification	0.8131	0.707	0.800

Table S4. Summary of the discriminatory power of several multivariate investigated in this study. *AUC – area under the curve of the receiver operating characteristic (ROC) curve, and their corresponding sensitivity and specificity values.*

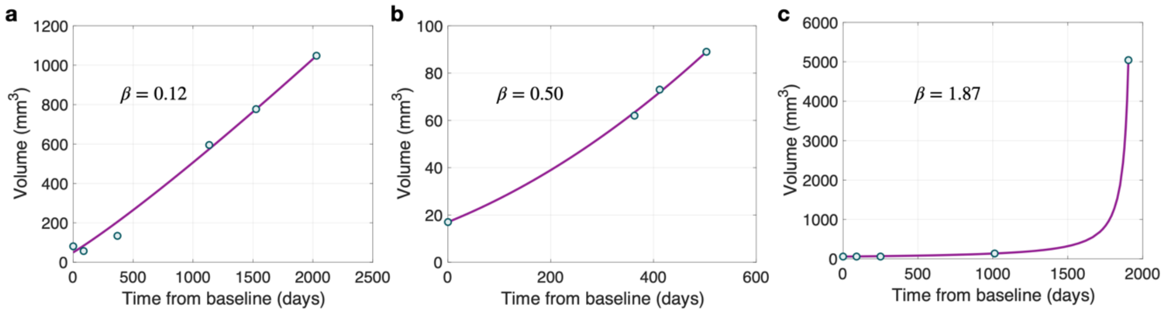

Figure S7. Figure adapted from Heuvelmans et al.[11] *where they concluded that lung cancer growth is best described by an exponential function. Blue points are obtained from Figure 3* [11] *using the software "WebPlotDigitizer" (https://automeris.io/WebPlotDigitizer/).*

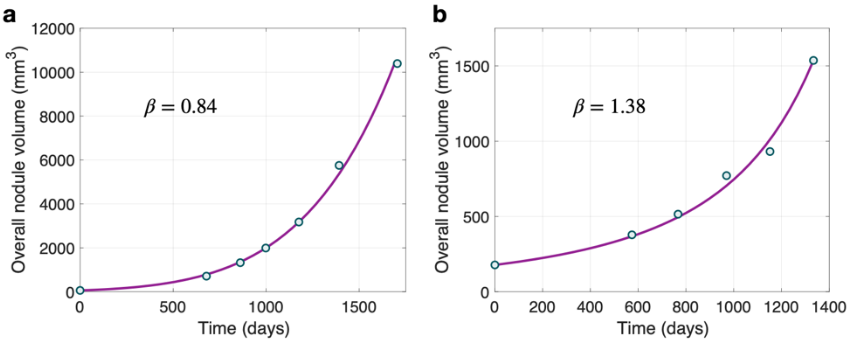

Figure S8. Figure adapted from de Margerie-Mellon et al.[13]*. Blue points are obtained from Figures 6-7 from the cited paper, were the authors stated that adenocarcinomas are best represented by exponential growth. The best curve fit was obtained for the values of β shown in the figure.*

a

β	Adenocarcinoma
< -0.1	35
-0.1 < beta < 0.1	1
0.1 < beta < 0.9	12
0.9 < beta < 1.1	5
> 1.1	69

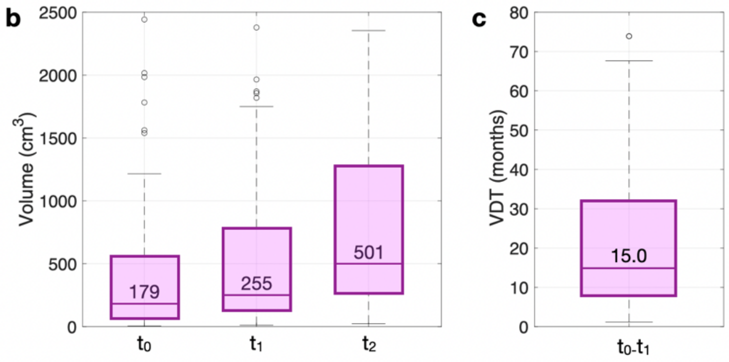

Figure S9. Evaluation of adenocarcinoma cases according to: a. *Types of growth according to the growth exponent* β***. b.*** *Evolution of volume over time.* ***c.*** *Volume doubling time (VDT).*
